# Comparative (Bio)monitoring of Airborne PAHs Using Mosses and Filters

**DOI:** 10.3390/molecules30194009

**Published:** 2025-10-07

**Authors:** Małgorzata Rajfur, Paweł Świsłowski, Tymoteusz Turlej, Oznur Isinkaralar, Kaan Isinkaralar, Sara Almasi, Arianna Callegari, Anca-Iulia Stoica

**Affiliations:** 1 Institute of Biology, University of Opole, 45-032 Opole, Poland; 2 Department of Power Systems and Environmental Protection Facilities, AGH University of Krakow, 30-059 Cracow, Poland; turlej@agh.edu.pl; 3 Department of Landscape Architecture, Faculty of Engineering and Architecture, Kastamonu University, 37150 Kastamonu, Türkiye; obulan@kastamonu.edu.tr; 4 Department of Environmental Engineering, Faculty of Engineering and Architecture, Kastamonu University, 37150 Kastamonu, Türkiye; kisinkaralar@kastamonu.edu.tr; 5 Department of Civil Engineering and Architecture, Faculty of Engineering, University of Pavia, 27100 Pavia, Italyarianna.callegari@unipv.it (A.C.); 6 Institute of Sanitary Engineering and Water Pollution Control (SIG), University of Natural Resources and Life Sciences, 1190 Vienna, Austria; anca.stoica@boku.ac.at

**Keywords:** air filters, atmospheric pollution, moss bioaccumulation, environmental monitoring, urban air quality

## Abstract

The present investigation provides a comparative six-month analysis of atmospheric pollution by polycyclic aromatic hydrocarbons (PAHs) in the urban region of Opole, Poland. The study employs dual monitoring methods: traditional quartz filter-based active air sampling and active moss biomonitoring using *Pleurozium schreberi*, *Sphagnum fallax*, and *Dicranum polysetum* mosses. The experimental campaign took place from August 2021 to February 2022, spanning the autumn and winter seasons. PAH concentrations were measured using gas chromatography–mass spectrometry (GC-MS) following methodical sample extraction protocols. Filters documented transient air changes in PAHs, particularly high-molecular-weight (HMW) components such as benzo[a]pyrene (BaP), which exhibited considerable increases during the colder months due to heightened heating activities and less dispersion. The size of particles deposited on the filters varied from 0.16 to 73.6 μm, with an average size of 0.71 μm. Mosses exhibited cumulative uptake trends, with *D. polysetum* showing the greatest bioaccumulation efficiency, particularly for low- and medium-molecular-weight PAHs, followed by *P. schreberi* and *S. fallax*. Meteorological indices, including sun radiation and air temperature, demonstrated significant negative relationships with PAH buildup in mosses. Diagnostic ratio analysis verified primarily pyrogenic sources (e.g., fossil fuel burning), although petrogenic contributions were detected in *D. polysetum*, indicating its increased sensitivity to evaporative emissions. The study shows that the integration of moss biomonitoring with traditional filter samples provides a strong, complementary framework for assessing air quality, particularly in fluctuating meteorological settings. The results advocate for the integration of moss-based methodologies into environmental monitoring initiatives and provide significant insights into contaminant dynamics influenced by seasonal and meteorological factors.

## 1. Introduction

In the domain of environmental quality monitoring, the use of biomonitoring, particularly with mosses, is a pivotal method for assessing atmospheric pollution, particularly concerning polycyclic aromatic hydrocarbons (PAHs) and heavy metals. Response to airborne pollutants, especially when analysing deposits of harmful substances, improves understanding of ecological health and environmental risks.

Mosses have been recognised for their capacity to accumulate heavy metals and PAHs, making them suitable choices for environmental monitoring. Research by Macedo-Miranda et al. highlights the effectiveness of mosses in tracking atmospheric deposition of heavy metals, documenting their use as bioindicators since the late 1960s [1].

The moss bag technique, which involves placing moss samples in bags across potential pollution sources, offers a cost-effective alternative to traditional air quality monitoring methods. Paoli et al. discuss the implications of using epiphytic lichens and mosses as indicators of air pollution in their research surrounding municipal waste landfills; these methods help elucidate the deposition of pollutants related to human activity [2]. The sensitivity of mosses to changes in air quality and atmospheric deposition rates is crucial for developing strategies to mitigate pollution’s effects. Moreover, this technique allows researchers to analyse spatial and temporal patterns of atmospheric trace elements, enhancing our understanding of how urban environments interact with environmental contaminants over time [3,4].

In the context of atmospheric pollution with PAHs, works by Burstyn et al. highlight the nature of understanding how these pollutants affect ecological health and human health outcomes, particularly in urban settings [5]. Through biomonitoring practices, quantification of PAHs can be more accessible, thereby facilitating interventions aimed at reducing exposure and enhancing environmental quality.

Importantly, studies on the relationship between atmospheric pollutants and biological response—like those of Stanojković et al.—emphasise that mosses are responsive bioindicators of both metal and organic compound contamination [6]. These insights underline the urgency of using living organisms for monitoring pollution levels, considering their capacity to reflect real-time ecological changes.

The integration of mosses in comprehensive air quality assessment frameworks is effective for generating actionable data across various contexts. Research findings indicate that traditional monitoring techniques—often resource-intensive—can benefit from the relatively low-cost and efficiency of biomonitoring practices [7]. Together with advanced techniques such as mass spectrometry and inferential analysis methods, the incorporation of biological indicators enriches the landscape of environmental monitoring, promising effective and holistic solutions to ongoing pollution challenges.

The impact of particle size on the monitoring of atmospheric pollution, particularly concerning PAHs, has garnered significant attention in recent research due to the distinct behaviour of PAHs in various airborne particle size fractions. This relationship is crucial for accurately assessing the environmental and health risks associated with PAH exposure, which is influenced by how these compounds interact with different sizes of particulate matter (PM).

Recent studies illustrate that PAHs exhibit size-dependent partitioning between gas and particle phases, impacting their pollutant behaviour and toxicity. For instance, a study by Pongpiachan shows that smaller particles in urban environments tend to associate with higher concentrations of PAHs, attributing this trend to their larger surface area and greater capacity for adsorbing these organic compounds [8]. This finding emphasises the importance of size segregation in monitoring efforts to obtain a clearer picture of atmospheric PAH distribution and its associated health risks.

The size segregation of PAHs is further explored by Liu et al., who discuss how environmental conditions influence the kinetics of atmospheric reactions involving PAHs distributed across diverse particle sizes [9]. They note that PAHs present in the bulk of larger particles may not interact readily with gas-phase oxidants, indicating that larger particles can behave differently concerning atmospheric reactions and thereby pose varying levels of risk over time, depending on the particle size. An understanding of these dynamics is essential when considering the design of monitoring strategies that seek to inform regulatory actions.

Research by Zhou et al. highlights the seasonal variations and size distributions of PAHs in atmospheric particles, particularly in medium-sized cities [10]. Their findings suggest that environmental factors, such as temperature and photochemical reaction intensity, significantly affect the particle size distribution of PAHs. For example, lighter PAHs tend to be present in the gas phase during warmer months, whereas larger, heavier PAHs are associated with finer particulate matter. This observation opens avenues for seasonal monitoring protocols that can adapt to these variations in PAH behaviour in conjunction with changing climatic conditions.

Kistaubayeva et al. have also examined how atmospheric PAHs tend to partition differently based on size, focusing on urban areas [11]. Their research indicates that carcinogenic PAH compounds, particularly those comprising larger molecules, are predominantly found in fine particle fractions (0.5–1 µm). This size range is concerning as these smaller particles can penetrate deeper into the respiratory system, influencing public health outcomes. Moreover, research by Khan et al. suggests that particulate size is a determining factor in PAH adsorption, highlighting the need for effective filtration materials for atmospheric sampling [12]. The study indicates that the efficiency of filters used in air quality monitoring is directly correlated to particle size, necessitating careful selection of filter types to optimise pollutant capture across the spectrum of particle sizes typically encountered in urban atmospheres.

The use of filters for monitoring PAHs in ambient air has involved techniques such as passive sampling with polyurethane foam (PUF) filters. Research indicates that PUF filters can effectively accumulate semivolatile organic compounds, including PAHs, from the atmosphere [13]. The technique allows for capturing both the vapour and particulate fractions of these pollutants, which is essential since PAHs can exist in different phases depending on environmental conditions such as temperature and humidity. The analysis of these samples typically utilises gas chromatography-mass spectrometry (GC-MS), which enables accurate detection and quantification of specific PAH compounds [14,15].

The characterisation of PAHs using filters is beneficial in diverse contexts, particularly urban environments where emissions from vehicles, industrial activities, and heating processes are prevalent. The study by Byambaa et al. investigated the sources and characteristics of PAHs in ambient total suspended particles in Ulaanbaatar City, Mongolia, using filter sampling methods [16]. These studies underscore how filters can capture a wide array of PAH compounds, allowing researchers to assess their contribution from various sources, which in turn aids in regulatory assessment and pollution management strategies. Moreover, filter-based monitoring facilitates longitudinal studies that are critical for understanding seasonal variations in PAH concentrations. For example, Zhang et al. reported significant differences in PAH levels during cold and warm weather months in Japan, revealing that combustion patterns change with seasons, affecting the atmospheric loading of these pollutants [17]. This temporal aspect is critical for urban planners and public health officials aiming to mitigate pollution exposure during peak exposure times.

Owing to the carcinogenic and mutagenic properties of PAHs, there is an urgent need to monitor these compounds in relation to human exposure implications. Studies like that of Amiri et al. highlight how various human activities, particularly in urban settings, contribute to PAH pollution, necessitating effective management of exposure and public health protection [18]. Implementing robust air quality monitoring mechanisms via filter analysis can thus serve as a first line of defence against potential health risks associated with PAH exposure. Furthermore, recent research has increasingly focused on utilising passive sampling methods that combine filters with field sampling devices to provide a more user-friendly and cost-effective solution for air quality monitoring. For instance, Adannou et al. emphasised the advantages of passive sampling in locations where traditional monitoring stations may not be feasible [19]. This flexibility allows for wider geographical coverage and continuous monitoring, which is essential for identifying pollution hotspots and informing remediation strategies.

The data obtained through filter-based monitoring can greatly enhance the understanding of geographical and temporal patterns of atmospheric PAHs. The distinctive profiles of PAHs associated with different pollution sources allow researchers to not only measure levels but also identify attribution from specific contributors, such as transport emissions, industrial discharges, or biomass combustion [14,16]. This specificity is vital for effective environmental policies aimed at reducing PAH emissions at their sources.

The novelty of this study lies in its direct and systematic comparison of two monitoring approaches—active biomonitoring with mosses and quartz fibre filter sampling—under identical urban conditions. While previous research has applied either moss biomonitoring or filter-based methods separately, to our knowledge, no study has combined them in a six-month campaign to assess their complementarity in monitoring airborne PAHs. Our results provide new evidence on species-specific differences in PAH accumulation (particularly for *Dicranum polysetum*), highlight the influence of meteorological drivers on moss bioaccumulation, and demonstrate how biological and instrumental techniques together deliver a more comprehensive and reliable assessment of urban air quality. This integrative perspective, bridging ecological indicators with conventional monitoring, represents a methodological advance that can inform both environmental science and applied air quality management.

The objective of this study was to evaluate the effectiveness of active moss biomonitoring as an alternative and complementary approach to the conventional filter-based method for assessing urban air pollution with PAHs. The study focused on comparing the bioaccumulation efficiency of three moss species (*Pleurozium schreberi*, *Sphagnum fallax*, and *Dicranum polysetum*) with quartz filter sampling under the same urban conditions, analysing the influence of meteorological factors such as air temperature and solar radiation on the accumulation of PAHs, and determining whether the integration of moss biomonitoring with filter-based sampling can provide a more comprehensive picture of PAH pollution dynamics in urban air.

## 2. Results and Discussion

### 2.1. Filters and Mosses

**Figure 1:**
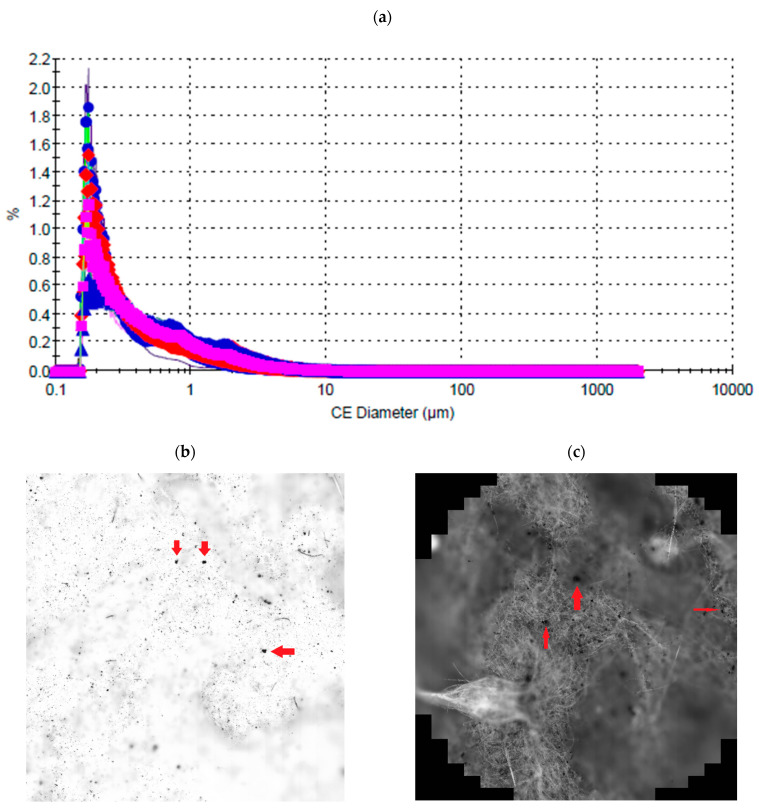
Examination of particles on filters: (**a**) particle size distributions for several selected filters; different curves represent data from different sampling days; (**b**) microscopic image of the surface of one filter showing deposited particles (50× magnification); (**c**) surface view of another filter with visibly denser particle deposition (50× magnification).

Figure 1 below shows the percentage of each particle size fraction deposited on the filters. As a result of the analyses, 132,574 particles of various sizes were identified/counted. Their range/scatter ranged from a minimum size of 0.16 μm to a maximum value of 73.59 μm. The average particle size value was 0.71 μm (Figure 1).

Figure 1 presents the results of the analysis of solid particles deposited on selected 47 mm membrane filters, performed using the Morphologi 4 automated image analysis system. Figure 1a illustrates the number-based particle size distributions, expressed as the equivalent circle diameter (CE Diameter, µm). The x-axis shows the particle diameter in micrometres, while the y-axis indicates the percentage of particles within each size range. The curves correspond to data obtained from several selected filters collected on different days, allowing for a comparative assessment of particle size characteristics across the sampling period. Figure 1b shows a microscopic image of one of the analysed filters, captured at 50× magnification. Deposited particles of varying shapes and sizes are visible on the surface of the membrane. Larger particles are marked with red arrows. Figure 1c presents the image of another filter, also at 50× magnification. In this case, the membrane appears darker, indicating a greater total deposition of particulate material. Larger particles are again marked with red arrows, as in Figure 1b.

The data presented in Table 1 provide an assessment of the variability in both the quantity and morphology of solid particles deposited on selected 47 mm membrane filters used in the conducted air quality measurements. The number of detected particles ranged from approximately 46,800 (Filter No. 43) to over 174,000 (Filter No. 34), reflecting differences in the concentration of airborne particulate matter on the respective sampling days. The mean particle size, expressed as the equivalent circular diameter (CE Diameter), varied between 0.42 µm and 0.90 µm, with the highest values observed for Filter No. 22. Morphological parameters such as circularity (HS Circularity), aspect ratio, elongation, solidity, and convexity reveal a broad range of particle geometries—from more compact and regular shapes to elongated and irregular forms. The observed morphological diversity may be attributed to the nature of particulate matter emission sources (e.g., fuel combustion, vehicular traffic, and industrial activity) as well as to atmospheric conditions that influence particle transport and deposition during the sampling campaign [20].

**Figure 2:**
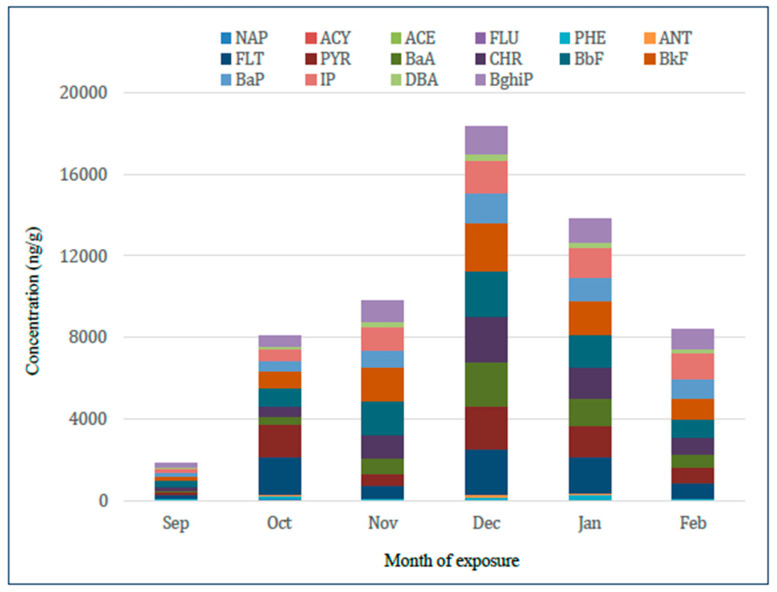
Monthly mean concentrations of PAHs (μg/g) measured using quartz air filters. The peak observed in December likely reflects increased emissions, possibly due to heating activities. The graph illustrates the wide range of monthly pollutant concentrations, reflecting fluctuations in emission intensity and meteorological conditions; Sep—September, Oct—October, Nov—November, Dec—December, Jan—January, Feb—February.

Quartz air filters were used to measure the monthly mean concentrations of 16 individual PAHs, as shown in Figure 2 below. The filter data reveal a distinct seasonal pattern, with the highest PAH concentrations recorded in December and January. This trend coincides with the heating season, during which increased emissions from domestic combustion and reduced atmospheric dispersion—caused by low temperatures and inversion layers—contribute to elevated pollution levels. The analysed compounds identified BaP, BbF, and IP as the main contributors to total PAH concentrations, all of which are commonly associated with combustion sources. The real-time ambient pollution levels captured by the filters confirm this seasonal variation.

The effect of particle diameter, measured in micrometres (μm), on deposited particles has been extensively investigated across various fields, highlighting a significant relationship between particle size and deposition efficiency within different environments, including biological systems and industrial applications. A robust body of literature reveals that increased particle diameter typically enhances deposition rates through various mechanisms, namely inertial impaction and gravitational sedimentation, depending on the airflow dynamics and the surface characteristics of the deposition target [21,22]. The relationship between particle size and deposition is also fundamentally tied to the environment in which particles are suspended. For instance, variations in ambient temperature and humidity substantially impact smaller particle sizes (sub-0.5 μm), emphasising the non-linear nature of deposition efficiency with environmental factors [23]. The calculations and empirical observations suggest a decrease in effective air quality, correlating with the presence of larger particulate matter in densely populated regions, which advocates for more stringent monitoring protocols [24,25]. Research has shown that the mean concentrations of PAHs in PM sampled on quartz air filters can vary widely depending on the sampling location, environmental conditions, and local emission sources. Studies conducted in urban areas typically show higher concentrations of PAHs compared to rural settings. For instance, extensive monitoring in urban and semi-urban regions, as well as a thorough investigation in Kumasi, Ghana, revealed mean PAH levels of 0.82 μg/g. to over 1.7 μg/g, stressing the need for continuous air quality assessments, with significant contributions from local traffic and industrial emissions [26]. The hazardous nature of PAHs necessitates a comprehensive understanding of their mean concentrations in air samples collected through quartz filters. Research indicates that mean values of total PAHs can range significantly across geographic regions, with reported values sometimes exceeding 100 μg/g in heavily industrialised areas. Geldenhuys et al. reported varying concentrations of particulate PAHs emitted during agricultural burning events, highlighting the localised effects of biomass combustion on PAH levels [27].

**Figure 3:**
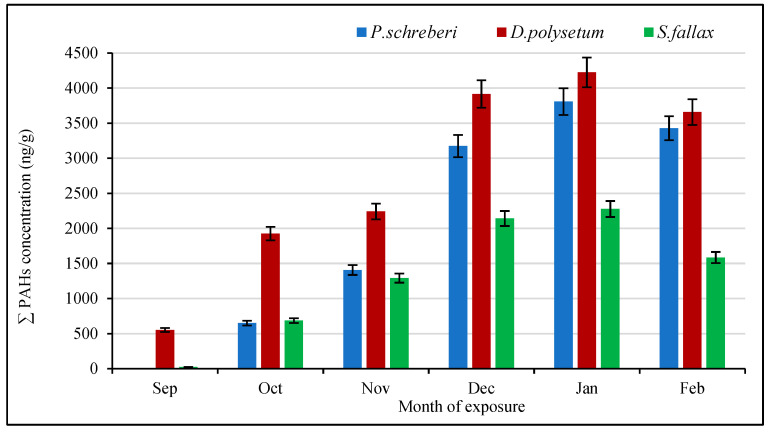
Monthly PAH concentrations (μg/g) measured during a six-month monitoring period in three moss species: *P. schreberi*, *D. polysetum*, and *S. fallax*, collected sequentially under an active biomonitoring approach.

**Figure 4:**
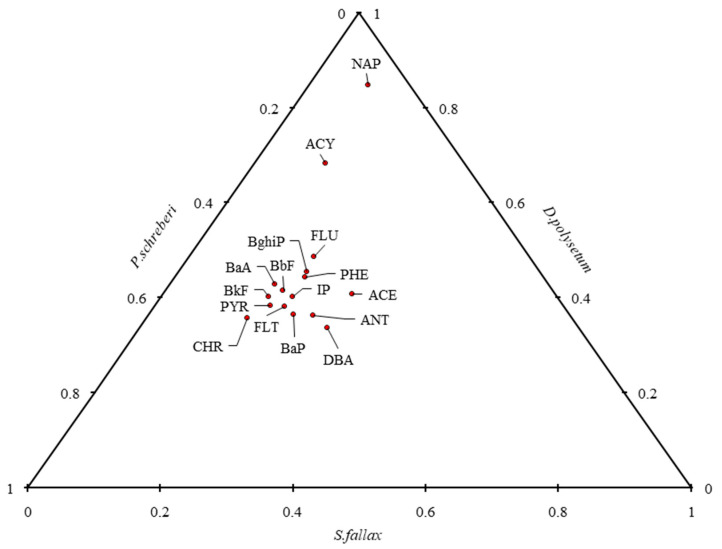
Ternary plot showing the relative contribution of three moss species to the summed concentration of individual PAHs. Dots represent the maximum concentration recorded for each PAH across all moss species.

The accumulation of the same PAH compounds in the moss species *S. fallax*, *D. polysetum*, and *P. schreberi* is shown cumulatively in Figure 3. The results indicate that mosses are effective bioindicators, capable of absorbing and retaining airborne PAHs over extended periods. The highest PAH concentrations in mosses were recorded during the winter months, consistent with seasonal increases in combustion-related emissions [28] as illustrated in Figure 3. This seasonal variation is also linked to lower photoreactivity and increased emissions from domestic heating sources, which enhance the persistence of PAHs in the environment [29]. The ternary plot in Figure 4 illustrates the distribution percentages between *S. fallax*, *D. polysetum*, and *P. schreberi,* which accumulate individual PAH compounds. The distribution analysis reveals that D. polysetum dominates the accumulation of NAP, ACY, and PHE, as well as other PAH compounds, due to its highest recorded concentrations of these compounds. The visual representation illustrates the diverse abilities of biological organisms to accumulate PAHs, supporting the results presented in Figure 3 and Figure 4. Studies by Rajfur et al. [30] support the findings that show different species display distinct patterns of PAH bioaccumulation under standardised exposure scenarios.

Among the studied species, *D. polysetum* exhibited the highest accumulation of both low-molecular-weight (LMW, e.g., NAP, ACY) and high-molecular-weight (HMW, e.g., BbF, BaP) PAHs. This is attributed to its favourable surface structure and wax composition, which enhance the adsorption of a broad range of compounds. In addition to surface structure, PAH uptake in mosses occurs through two main pathways: gaseous absorption and particulate deposition. Moss leaves create an interface with polluted air and water, making them effective sites for the accumulation of PAHs [31]. Mosses are recognised for their ability to accumulate atmospheric pollutants, particularly organic compounds like PAHs, which can stem from both natural and anthropogenic sources, including combustion processes and industrial activities [32]. Studies indicate that *P. schreberi* is particularly effective as a bioindicator, accumulating up to 55% more PAHs than *D. polysetum* and 25% more than *S. fallax* during long-term monitoring [30]. This finding is corroborated by additional research, which highlights that *P. schreberi* can selectively accumulate various PAH compounds, reflecting its high potential for monitoring atmospheric pollution [33]. The differences in accumulation among moss species are not only due to morphological traits but also to physiological adaptations. Living mosses can dynamically regulate the uptake rates and metabolic processing of PAHs in response to environmental stress, thereby enhancing their resilience against toxic compounds [33]. The differences in accumulation among these moss species can be attributed to their physiological and ecological characteristics [34]. Investigations into the specific PAH profiles accumulated by each moss species reveal that the most commonly detected PAH compounds include phenanthrene, anthracene, and pyrene. The hydrophobic properties of these organic pollutants enhance their retention within the porous structure of mosses, promoting sustained bioaccumulation over time [32]. Moreover, PAHs with higher partition coefficients and slower degradation rates accumulate more readily in moss tissues, driven by their affinity for organic carbon and lipid membranes present in moss cells [31,35]. The presence of high-molecular-weight PAHs, known for their carcinogenic and mutagenic properties, raises concerns about their impact on both environmental and human health [36]. Recent studies also emphasise that mosses, in addition to indicating environmental contamination, may accumulate PAHs in a way that links to public health. Acting as natural filters, they can retain particulate matter, suggesting the need for further research connecting PAH levels in moss with human health biomarkers [5,16].

**Figure 5:**
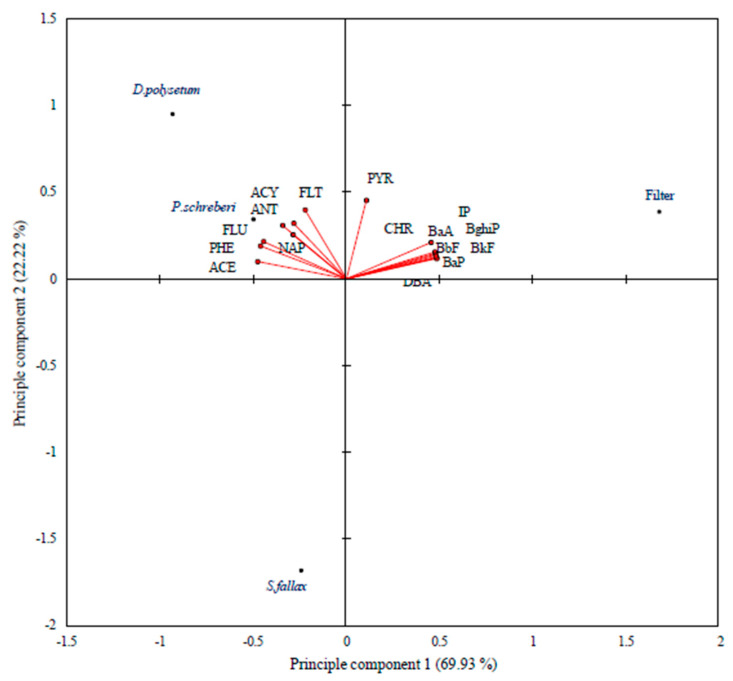
Principal component analysis illustrating the relationship between PAH concentrations and moss species and filter.

The Principal Component Analysis (PCA) depicted in Figure 5 illustrates the correlations between the selected PAH concentrations and quartz filter measurements, as well as the moss species. The two principal components successfully represent the original dataset, accounting for 92.1% of the total variance (PC1: 69.93%, PC2: 22.22%). The upper left position of *D. polysetum* indicates its affinity for accumulating the lighter PAH compounds NAP, ACY and PHE. *P. schreberi* appears near the centre of the PCA plot, yet *S. fallax* sits in the lower quadrant, which indicates that both moss species demonstrate different affinities with the majority of PAH compounds. Moss samples showed relatively higher concentrations of LMW PAHs, likely due to their prolonged atmospheric residence time and enhanced tissue penetration. The research [30] shows that *D. polysetum* shows superior light-weight and medium-weight PAH accumulation, particularly for NAP, PHE and FLT. The quartz filter occupies the most distant position on the right side because it selectively traps heavy-weight PAHs, including BaP, BghiP, IP, BbF, and BkF. Compared to quartz filters, which reflect short-term monthly variations, mosses provide a longer-term record of atmospheric PAH deposition. Filters more effectively captured HMW PAHs, which are primarily associated with particulate matter. The results from PCA analysis validate that moss samples and filters effectively cooperate and complement each other in measuring airborne PAH pollution.

**Figure 6:**
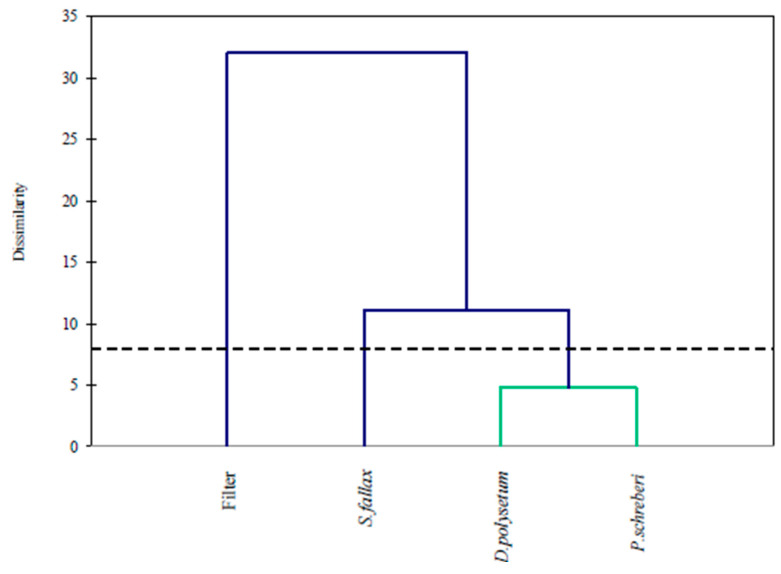
Cluster analysis of PAH concentrations in three moss species and a filter.

The hierarchical cluster analysis results in Figure 6 demonstrate how moss species and quartz filter specimens group according to their parallel patterns of PAH accumulation. The dendrogram shows that quartz filter data form an independent cluster, which stands apart from every moss species data. The filter exhibits dissimilar accumulation patterns compared to all biological materials, as a result of this separation. The active air sampling nature of the filter enables it to capture both gaseous and particulate-bound PAHs and high-molecular-weight compounds, as shown in Figure 5 (PCA). The accumulation patterns of light- to mid-weight PAHs match between the moss species *D. polysetum* and *P. schreberi*. The accumulation profile of *S. fallax* stands out from that of other mosses based on phylogenetic analysis, as it shows less efficiency in retaining PAHs, according to PCA.

The study of air filters, particularly dust collectors, and their sorptive properties offers valuable insights into material–particulate interactions. This analysis compares conventional filtration media with mosses, highlighting their distinct mechanisms for absorbing pollutants and moisture. The efficiency of these systems varies depending on their structural and chemical characteristics. Traditional air filters rely on physical and mechanical processes to trap particulates. In an evaluation study, static filtration systems were reported to achieve a high dust collection efficiency of up to 99% in various industrial applications, including environments with high dust concentrations [37]. This high efficiency stems from the filters’ design that maximises the surface area available for dust collection while allowing air permeability conducive to function without excessive pressure drops [38]. By contrast, mosses combine physical trapping with biological and ecological processes. Their porous architecture and hygroscopic properties not only capture airborne particles but also provide conditions for biochemical interactions that enhance retention of organic pollutants such as PAHs [32,39]. In contrast, mosses utilise a biological and ecological approach for dust retention and sorption. Research indicates that the structure of mosses, particularly their porous architecture and hygroscopic properties, enables them to capture not only airborne dust but also to retain moisture from the atmosphere [40,41]. This physically intertwined matrix of stems and leaves provides a significantly larger surface area relative to their volume compared to conventional synthetic air filters. The sorptive capacity of mosses can thus be material-specific, involving complex biochemical interactions that contribute to their ability to capture pollutants. The clustering structure supports the conclusion that moss species differ in their accumulation efficiency and specificity and that the moss-filter contrast is substantial enough to treat them as complementary, not interchangeable, tools for PAH monitoring [33]. This perspective is echoed, which highlights the value of using mosses alongside classical filters in urban air quality assessments. The study results demonstrate a steady increase in PAH concentrations over six months of monitoring across all species, due to continuous pollutant deposition and absorption patterns. *D. polysetum* demonstrated the most efficient absorption capabilities for almost all tested compounds, as it showed superior performance in capturing HMW PAHs. *P. schreberi* accumulated PAH compounds at an intermediate rate, but *S. fallax* kept showing minimal absorption patterns, which supported earlier evidence that this species possesses lower trapping efficiency. Overall, the biological mechanisms underlying moss accumulation of PAHs are complex, involving both chemical properties of PAHs and physiological adaptations of moss species [31,33]. This highlights the importance of species-specific studies in identifying the most effective bioindicators under various environmental conditions [5,42]. The combined method of pollutant monitoring reveals important information about moss behaviour during long-term contact with pollutants, while providing better insight into environmental pollutant distribution patterns [43].

Table 2 summarises the cumulative concentrations of PAHs recorded in the moss species and the monthly means obtained from quartz filters. Results highlight the varying bioaccumulation capacities among mosses, allowing for comparison with conventional air filters under the same environmental conditions. Among the mosses, *D. polysetum* showed the highest accumulation capacity for most compounds.

The mosses demonstrated reliable uptake over extended periods, which enabled their use for integrated time-based monitoring, despite the filters capturing larger absolute amounts, particularly for high-molecular-weight PAHs. The analysis of filter and moss data provides complete information about short-term and long-term atmospheric PAH trends. Cumulative accumulation graphs for selected PAHs in mosses are presented in Supplementary Materials (Figure S1).

### 2.2. Meteorological Influences on PAH Accumulation

The influence of meteorological conditions on the accumulation of PAHs was examined through both regression analysis (Table S1 in Supplementary Materials) and monthly parameter monitoring (Table S2 in Supplementary Materials). Among the tested parameters, solar radiation and air temperature (maximum, minimum, and mean) showed significant linear relationships with PAH concentrations, particularly in moss species.

During the six-month observation period, linear regression models showed the relationships between meteorological factors and total PAH concentrations found in the quartz filter and three moss species. The moss *D. polysetum* exhibited the strongest positive correlations among all studied species, as it reacted strongly to solar radiation and mean temperature fluctuations (y = 4834x − 346 and y = 4282x − 223). Temperature and solar radiation influenced *P. schreberi* similarly, but its response coefficients were slightly lower. *S. fallax* recorded correlations that indicated its accumulation response was moderately stable but unreactive to environmental changes. Filter-based measurements of PAHs did not show a relationship with meteorological variables. The analysis of filters over 24 h only reveals current atmospheric conditions, rather than long-term accumulation effects, due to their limited duration. The monthly meteorological variation (Table S2) exhibits a clear seasonal trend. The duration from September to February resulted in significant reductions in solar radiation and temperature, with their minimum points occurring in December and January. Higher PAH concentrations in mosses reached their peak in December, coinciding with increased residential heating activities and limited atmospheric dispersion capabilities [44]. Mosses demonstrate superior suitability as indicators of long-term climatic effects on PAH deposition due to their ability to accumulate pollutants from the environment. Higher temperatures often lead to increased volatilisation of particulate-bound PAHs into the gas phase, which enhances their transport and biological uptake by organisms [45,46]. Winters reveal the highest pollution quantities because emissions combine with meteorological stagnation, whereas uptake rates increase under warm and sunny weather conditions [47,48]. Moreover, wind speed is particularly pivotal in the dispersion of atmospheric PAHs. Increased wind speed can facilitate the dilution of PAHs, while lower wind speeds may lead to localised accumulation and higher exposure levels [48,49].

Solar intensity exhibits an inverse relationship with PAH accumulation throughout the six-month observation period, as indicated by visual findings. All moss species accumulated less PAHs during solar-rich September and October, whereas their concentrations rose substantially during the dark and cold winter months, particularly December and January. The decrease in solar radiation appears to reduce photodegradation of PAHs, thus enabling their atmospheric survival before mosses absorb them. The concentration levels of *D. polysetum* responded most strongly when solar radiation decreased. The uptake patterns in *P. schreberi* were similar to those of *S. fallax*. Still, they exhibited reduced intensity, whereas D. polysetum demonstrated the strongest response to changes in solar radiation (Figure S2 in the Supplementary Materials). The chart highlights the inverse relationship between solar intensity and pollutant accumulation, suggesting lower degradation and increased PAH deposition during months with reduced solar radiation. Solar radiation plays a crucial role in degrading PAHs, particularly for low- and medium-weight compounds [50]. This finding supports the previously observed negative correlation. PAH concentrations rose in this study because photolysis decreased and emissions increased when radiation levels were low [51].

**Figure 7:**
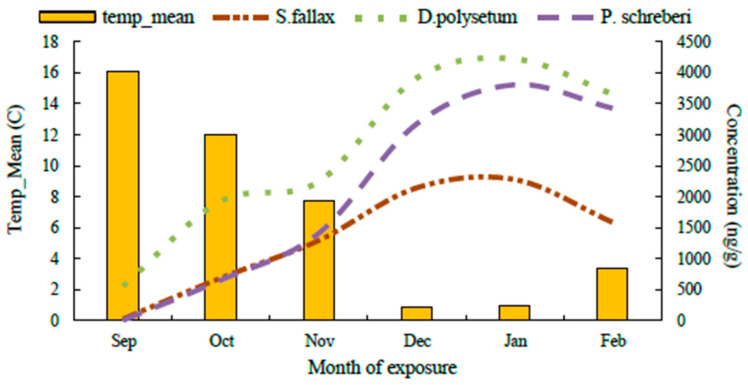
Combined diagram showing the relationship between mean temperature (°C) and cumulative PAH concentrations (μg/g) in three moss species over six months. Yellow bars represent monthly mean temperatures, while the lines indicate cumulative PAH accumulation in *S. fallax*, *D. polysetum*, and *P. schreberi*.

Temperature data shows a direct connection with PAH concentrations as the accumulation reaches its peak in these colder months, according to Figure 7. The maximum and minimum temperatures developed negative relationships with PAH accumulation levels. The data indicate that the minimum temperature (Figure S3 in the Supplementary Materials) creates a stronger relationship, as PAH levels increased during nights with lower temperatures in January and February. Nighttime conditions favour the settlement of pollution on moss surfaces due to minimal atmospheric turbulence, along with reduced volatilisation rates [52].

The inverse relationship between temperature and PAH accumulation suggests that lower temperatures may favour pollutant deposition and reduce volatilisation or degradation [53,54]. Overall, the data support the conclusion that colder conditions—marked by lower solar radiation and temperature—are conducive to greater PAH deposition. These results reinforce the suitability of mosses as bioindicators that are sensitive to both chemical loads and meteorological variations [55].

### 2.3. Source Identification and Toxicity of PAHs

The diagnostic ratios for selected PAH compounds used to detect contamination sources appear in Table 3. The evaluation of these ratios depends on LMW and HMW PAH abundances, as well as FL/(FL + PYR) and IP/(IP + BghiP) and BaA/(BaA + CHR) compound pairs. Ratios such as Phenanthrene/Anthracene and Fluoranthene/Pyrene have consistently been referenced to delineate source origin in various studies, establishing a methodological framework for pollutant attribution [56]. According to the literature, an FL/(FL + PYR) ratio greater than 0.5 typically indicates pyrogenic sources, such as fossil fuel combustion. Similarly, IP/(IP + BghiP) values above 0.5 are associated with diesel vehicle emissions, while BaA/(BaA + CHR) values greater than 0.35 suggest combustion-related origins. In contrast, a ΣLMW/ΣHMW ratio above 1 implies petrogenic sources, such as oil evaporation, whereas values below 1 indicate pyrogenic dominance. Lower molecular weight PAHs (LMW) are typically associated with biomass burning, while higher molecular weight PAHs (HMW) indicate more complex combustion processes, such as those resulting from fossil fuel burning [57]. Incorporating meteorological data such as temperature and precipitation, researchers were able to contextualise the deposition patterns of PAHs, thus enhancing the reliability of the source apportionment results derived from diagnostic ratios [29].

The filter data consistently demonstrate low ΣLMW/ΣHMW ratios averaging 0.03, which suggests combustion processes are the primary source of pollution. The vehicle exhaust signatures from diesel emissions can be confirmed by diagnostic ratios FL/(FL + PYR) (average: 0.52) and IP/(IP + BghiP) (average: 0.55) because they consistently exceed 0.5 [58]. The moss *S. fallax* exhibited an even distribution of LMW and HMW compounds, as indicated by its ΣLMW/ΣHMW ratios, which averaged 0.37, along with fluctuating diagnostic metrics. The ratios of FL/(FL + PYR) and IP/(IP + BghiP) measured at 0.53 and 0.51, respectively, indicated that the moss samples received PAH compounds from combinations of oil-related petrogenic and combustion-based pyrogenic sources. The analysis of *D. polysetum* revealed exceptionally high ΣLMW/ΣHMW ratios (3.06), as the moss accumulated a significant amount of low-molecular-weight PAHs, including NAP and ACY. The moss’s high capacity for gaseous airborne compounds implies petrogenic sources, which could stem from automotive evaporative emissions or local traffic activities. The diagnostic ratios from *P. schreberi* indicated a pyrogenic origin of contaminants, with a ΣLMW/ΣHMW ratio of 0.26. These findings matched what was observed through filter analysis. The mosses, particularly *D. polysetum*, demonstrated the ability to gather diverse sources beyond traffic emissions, which influenced both filter- and moss-based sampling results. The coexistence of multiple sources in urban environments often complicates the straightforward attribution of PAH sources to specific diagnostic ratios, necessitating the use of multivariate statistical methods in conjunction with traditional ratio analyses for more comprehensive assessments [59].

Based on the calculated *TEQ*s, the cumulative toxicity of PAHs relative to BaP can be set in the following descending series with cumulative concentrations of 16 individual PAHs (ng/g): 3842 (filters) > 239 (*P. schreberi*) > 236 (*D. polysetum*) > 116 (*S. fallax*). In contrast, the sum of PAHs considered carcinogenic (cPAHs) is as follows (ng/g): 6229 (filters) > 490 (*D. polysetum*) > 479 (*P. schreberi*) > 213 (*S. fallax*). The results presented here clearly indicate the differences previously discussed and presented regarding the accumulation of filters and mosses. It also confirms previous reports. The results obtained from active biomonitoring using mosses are not made less significant by this. Still, it should be remembered that these results are due to the biological activity of the organisms, which have different mechanisms for absorbing pollutants. The use of filters as part of conventional air monitoring yields standardised results, but we are not always able to use such devices and rely on their reports. In contaminated environments, the application of the *TEQ* method has been demonstrated to be crucial for identifying potential health risks associated with PAH exposure. For instance, Zhang et al. calculated BaP equivalents for PAHs present in various ecosystems, yielding significant insights into the potential toxicological risks [60]. *TEQ* assessment has also shown versatility in diverse geographical contexts. In regions experiencing transboundary pollution, PAH *TEQ* evaluations in mosses can provide crucial insights into long-range transport mechanisms of pollutants [32]. The utilisation of mosses as integrative environmental indicators, coupled with *TEQ* methodologies, thereby supports the establishment of global health risk databases for PAH exposure, reflecting environmental quality across diverse ecosystems [61]. Notably, assessments addressing the *TEQ* of PAHs in mosses have highlighted the necessity of continual monitoring for both environmental and human health implications [62]. For instance, studies have indicated alarming *TEQ* levels correlated with industrial activities, emphasising the need for heightened monitoring and methodological advancements in future research [63].

In summary, the novelty of this research is twofold. First, it presents a direct comparison between moss-based and filter-based monitoring approaches, revealing their complementary rather than interchangeable roles in assessing PAHs. Second, it identifies species-specific accumulation capacities and their dependence on meteorological variables, thereby extending current knowledge on the ecological functioning of mosses as bioindicators. These findings offer a new framework for integrating low-cost biomonitoring with established instrumental techniques in urban air quality surveillance.

## 3. Materials and Methods

### 3.1. Study Area

The research took place in the Opole municipality, located in southwestern Poland. The city of Opole is situated at 50°40′ N latitude and 17°55′ E longitude, near the Oder River, which influences its local environmental and climate characteristics [64]. As the capital of the Opole Voivodeship, Opole maintains its position as a mid-sized urban centre that divides its space between residential areas, industrial facilities and institutional buildings.

Opole operates under a climate system classified by Köppen, which combines warm temperate weather with transitional conditions from the Atlantic and continental air flow patterns. The area exhibits distinct differences in its seasonal weather patterns. The climate of Opole features summer temperatures ranging from 20 °C to 25 °C from June to August, while winter temperatures (December to February) remain below the freezing point. Annual precipitation levels in the region remain moderate, while summer rains are slightly heavier, making the area suitable for a humid climate designation [64].

**Figure 8:**
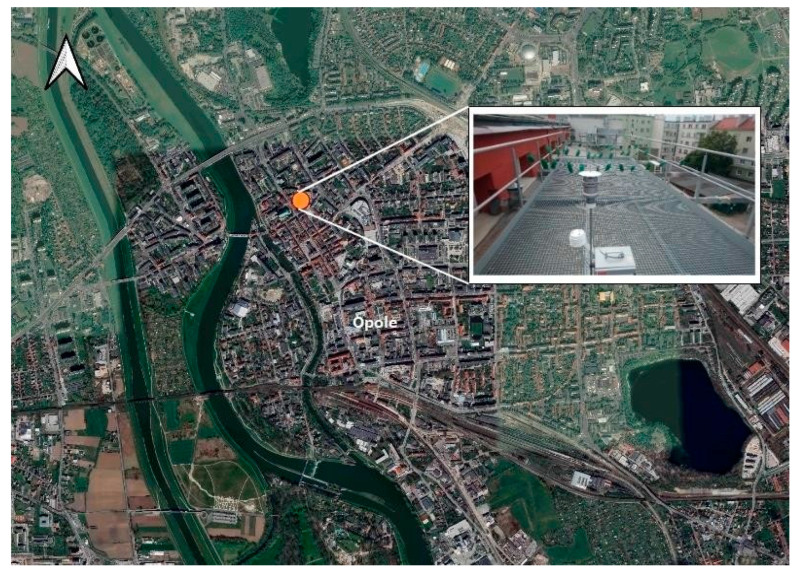
Location of the active air sampling site in the city of Opole, southwestern Poland. The orange dot marks the precise location of the sampling station, situated on the second-floor viewing platform of a faculty building at the University of Opole (50°40′15.23″ N and 17°55′17.72″ E).

The sampling site was located on the second floor of a building within the University of Opole faculty, situated at 50°40′15.23″ N and 17°55′17.72″ E (Figure 8). The elevated, unobstructed position allowed for perfect outdoor urban air exposure because researchers chose this spot to enable simultaneous biological and instrumental air quality measurements. The sampling period took place during the fall and winter seasons because this time frame overlaps with heating season conditions that enhance domestic fuel emissions, as well as atmospheric inversions that raise levels of PAHs in outdoor air.

### 3.2. Materials

QM-A quartz filters (Whatman, Maidstone, UK), 47 mm in diameter, were used to collect airborne particulate matter (PM). They offer low background contamination and excellent efficiency in capturing fine particles, ensuring accurate detection of PAHs in ambient air samples [65,66].

Three moss species were used for active biomonitoring purposes, including *P. schreberi*, *S. fallax*, and *D. polysetum*. The mosses came from a pollution-free forest region in the southeastern Polish region of Świętokrzyskie Voivodeship. Active biomonitoring samples from this region are commonly used for reference purposes due to the minimal industrial presence and geographical distance to pollution sources, as recommended by both the ICP Vegetation protocol and multiple past studies [30,67].

### 3.3. Methodology

The sampling process included one unused filter for background assessment and a pre-exposure control to verify the absence of contamination. The research spanned from 21 August 2021 to 21 February 2022 (six months), which resulted in 24 h filtration times for each of the collected filters. The PM10 high-volume air sampler (PNS3D15/LVS3D) performed sampling at a flow rate of 2.3 m^3^/h during the process. The sampler employs a quartz fibre filter as its core component to collect suspended particulate matter through air intake, thus trapping fine and coarse particles for chemical evaluations [68]. The sampling method adhered to EN 12341:2014 gravimetric standard from the European Committee for Standardization (CEN) for determining PM_10_ mass concentrations in ambient air [33].

The active biomonitoring involved the use of *P. schreberi, S. fallax*, and *D. polysetum* moss species due to their ability to accumulate PAHs and their specific physiological traits [30]. Moss specimens were obtained from the Świętokrzyskie Voivodeship background forest area and processed according to the ICP Vegetation methodology [69]. The assembly process included placing two grams of fresh moss into each nylon mesh bag. The researchers collected each sample from each species according to established dates throughout the monthly cycle. The unexposed control samples served to determine the initial background levels for all species. The analysis was conducted twice for each moss sample to verify its consistency [67].

Once all samples had been collected and transported to the laboratory, the moss and filter samples were dried at room temperature. The moss samples were then ground in an agate mortar, sieved through a 100-mesh sieve, and finally homogenised in a mixer for 10 min. A detailed description of the procedure for preparing moss and filter samples for measuring PAHs, as well as the analysis method using the GC-MS device, can be found in the supplementary document (Table S3 in Supplementary Materials). Calibration was performed using PAH-Mix 9 as an external standard, and PAH- Mix 24 (deuterated PAHs) was added to each sample before extraction to serve as an internal standard and correct for matrix effects. A nine-point calibration curve was generated for each one of the studied PAHs, and the linear correlation coefficient was higher than 0.9990 for all calibration curves. The accuracy of the method and reproducibility of the results were tested by different procedures: by a one-time per year laboratory test involved in a national round-robin test/proficiency test; by calibration before each measurement to compensate for any changes in measuring conditions during the measurement; and by (extracted) double determination of a blank value and a control sample. PAH diagnostic ratios were used to identify PAH emission sources from the literature [70]. The choice of elements and PAHs determined relates to compounds that were analysed in mosses by other authors [71,72]. For the PAHs determined, we used specific abbreviations of their names in the rest of the article: Naphthalene: NAP, Acenaphthylene: ACY, Acenaphthene: ACE, Fluorene: FLU, Phenanthrene: PHE, Anthracene: ANT, Fluoranthene: FLT, Pyrene: PYR, Benzo[a]anthracene: BaA, Chrysene: CHR, Benzo[b]fluoranthene: BbF, Benzo[k]fluoranthene: BkF, Benzo[a]pyrene: BaP, Dibenz[a,h]anthracene: DBA, Benzo[ghi]perylene: BghiP, and Indeno[1,2,3-cd]pyrene: IP [73].

As part of the study, an analysis of the particle size distribution was performed on 47 mm filters used to collect airborne particulate matter from daily ambient air samples. The measurements were carried out using the Morphologi 4 automated particle imaging system (Malvern Panalytical, Malvern, UK), which enables high-resolution light microscopy combined with advanced digital image processing for detailed assessment of particle size and shape [74].

Statistical analysis was conducted on a number-based basis, allowing for quantitative representation of the particle population structure independently of particle mass or volume. All measurements were fully automated and performed under fixed optical settings, segmentation parameters, and acquisition protocols, ensuring methodological consistency and enabling direct comparison of results obtained across successive filters.

Each filter was mounted in a dedicated 47 mm Filter Plate holder. For each sample, images were acquired from multiple distinct measurement areas located in various regions of the filter surface. This approach minimised the influence of local heterogeneity in particle deposition and ensured that the resulting data were representative of the entire filter. The area of each measurement field was 4 mm^2^.

Imaging was conducted using a 50× objective lens, enabling detection of particles with an equivalent circular diameter (CE diameter) in the range of 0.5 µm to 50 µm. To maintain image sharpness across the full depth of field, a Z-stacking routine was applied, comprising four layers above and two layers below the primary focal plane. The illumination was set to episcopic (top-light) mode in bright-field configuration, with manual adjustment of light intensity to match the optical characteristics of each filter surface.

Particle segmentation was performed using the Low Contrast algorithm, which proved effective in distinguishing particles from the background under low-contrast conditions—particularly relevant for heavily loaded filters bearing substantial quantities of deposited particulate matter. This method was adopted as the default for all analyses due to its robustness under typical measurement conditions. For quality control purposes, a limited number of samples were also analysed using manual thresholding to verify segmentation accuracy; however, the data obtained via this approach were not included in the statistical evaluation.

Particles were characterised in terms of size and shape, with parameters including CE diameter, length, width, perimeter, projected area, circularity, elongation, convexity, and solidity. For each filter, between 45,000 and 175,000 particles were registered, providing a detailed statistical basis for reconstructing size distributions and ensuring high representativeness of the results.

### 3.4. Calculations and Statistics

PAH diagnostic ratios obtained from the literature were employed to identify potential sources of PAH emissions [70]. Additionally, Toxic Equivalency Quotient (*TEQ*) allows the conversion of the total toxicity of PAHs to BaP according to the following formula:

$${TEQ}_{BaP}=\sum (C_{i}\times {TEF}_{i})$$

where

- *C_i_*: concentration of a given PAH
- *TEF_i_*: equivalent toxicity coefficient for a given compound [75]

Carcinogenic PAH (cPAH) Concentration, the sum of PAHs considered carcinogenic, was also calculated.

Statistical analyses were conducted using Microsoft Excel 2021 and STATISTICA software (version 13.3) for data processing and visualisation. Descriptive statistics included basic statistical parameters (such as minimum, maximum, and mean values) for each analysed PAH and meteorological condition indicator. The normality of data distribution was assessed using the Shapiro–Wilk test, which was selected due to the sample sizes [76]. A significance level of *p* < 0.05 was applied to reject the null hypothesis. The influence of meteorological conditions on the accumulation of PAHs was examined through regression analysis. Principal component analysis (PCA) was performed to explore associations between the measured PAHs and moss species and filter. A ternary diagram was employed to visualise the distribution percentages of individual PAH compounds accumulated by different moss species. This type of diagram illustrates the relationship among the three components by plotting their relative proportions [77]. Data were also clustered by calculating the Euclidean distances between each PAH distribution pattern (moss and filters) and clustering PAHs using a hierarchical single-linkage method (Statistica ver. 13.3).

## 4. Limitations and Future Perspectives

While the present study provides important insights, some limitations should be acknowledged. The monitoring was limited to a specific period and location, and no additional validation experiments were performed. Additionally, three moss species were tested, and further research is needed to validate their performance in various climatic and pollution contexts. Future work should extend the monitoring to multi-year campaigns, include additional bioindicator species, and integrate moss biomonitoring with health-relevant exposure indicators. It should also incorporate controlled validation tests. Such efforts will further strengthen the applicability of combined biomonitoring–filter approaches in environmental assessment and policy development.

## 5. Conclusions

This study shows the effectiveness of integrating moss biomonitoring and quartz fibre filter sampling in assessing atmospheric polycyclic aromatic hydrocarbon (PAH) pollution in urban environments. Mosses, particularly *D. polysetum*, proved to be efficient bioindicators for long-term accumulation of low- and medium-molecular-weight PAHs. At the same time, filters were more effective in detecting high-molecular-weight PAHs such as benzo[a]pyrene during winter. Seasonal variations in pollutant levels were primarily driven by temperature, solar radiation, and emissions related to combustion. Diagnostic PAH ratios identified both pyrogenic and petrogenic pollution sources.

The results emphasise that mosses and filters differ not only in the PAH fractions they capture, but also in the temporal resolution of monitoring: mosses integrate cumulative pollution over extended periods, whereas filters reflect short-term fluctuations and provide high-resolution chemical detail. PCA and cluster analyses confirmed this divergence, with filters forming a distinct group separated from mosses, highlighting their different accumulation mechanisms.

The advantages of moss biomonitoring include low cost, passive sampling, and the ability to reflect both gaseous absorption and particulate deposition over long timescales. Mosses are also sensitive to environmental drivers such as humidity and temperature, which makes them valuable ecological indicators. However, limitations include species-specific variability in accumulation efficiency, possible physiological responses that affect uptake, and lower quantitative precision compared to filters. Quartz filters, by contrast, are standardised and reproducible, highly efficient at trapping particulate-bound HMW PAHs. Yet, they are resource-intensive, limited to shorter exposure windows, and may underestimate the integrated pollutant burden that mosses naturally capture.

The combined use of biological and instrumental methods provides complementary insights into spatial and temporal patterns of pollution. Therefore, moss biomonitoring and quartz fibre filters should be regarded as complementary rather than interchangeable tools, and their integration offers the most comprehensive understanding of PAH distribution in the atmosphere.

## Figures and Tables

**Table 1:** Overview of quantitative and morphological characteristics of particles deposited on 47 mm membrane filters, including total particle counts, mean particle size, and selected shape descriptors derived from image analysis.

Filter ID	# Particles	CE Diameter Mean (µm)	HS Circularity Mean	Aspect Ratio Mean	Elongation Mean	Solidity Mean	Convexity Mean
Filter No. 1	166,480	0.83	0.504	0.701	0.299	0.864	0.877
Filter No. 4	76,473	0.59	0.443	0.685	0.315	0.840	0.861
Filter No. 7	93,646	0.54	0.407	0.670	0.330	0.823	0.849
Filter No. 10	123,955	0.75	0.461	0.690	0.310	0.847	0.864
Filter No. 16	171,365	0.42	0.326	0.651	0.349	0.789	0.834
Filter No. 19	80,527	0.83	0.483	0.657	0.343	0.848	0.882
Filter No. 22	70,715	0.90	0.570	0.698	0.302	0.887	0.903
Filter No. 25	105,059	0.72	0.458	0.692	0.308	0.849	0.857
Filter No. 28	57,855	0.51	0.460	0.660	0.340	0.834	0.889
Filter No. 31	127,364	0.56	0.474	0.678	0.322	0.843	0.877
Filter No. 34	174,523	0.44	0.387	0.670	0.330	0.814	0.850
Filter No. 37	130,610	0.51	0.531	0.711	0.289	0.897	0.904
Filter No. 40	142,715	0.83	0.549	0.713	0.287	0.900	0.909
Filter No. 43	46,827	0.86	0.534	0.662	0.338	0.883	0.932
Filter No. 52	132,574	0.71	0.537	0.628	0.372	0.873	0.953

**Table 2:** Cumulative concentrations of 16 individual PAHs (ng/g) in three moss species and the mean values from quartz filters over six months.

Method/PAH	NAP	ACY	ACE	FLU	PHE	ANT	FLT	PYR	BaA	CHR	BbF	BkF	BaP	IP	DBA	BghiP
**Filters**	33.8	11.1	5.18	8.59	124	34.3	1244	1139	908	1099	1267	1293	846	1061	197	853
***S. fallax***	36.4	6.00	13.9	15.04	334	23.0	550	292	28.6	91.2	60.3	37.0	21.4	31.9	10.6	33.8
***D. polysetum***	348	37.8	19.9	38.87	753	33.6	1067	646	77.4	213	141	92.1	35.6	64.9	12.6	79.3
***P. schreberi***	25.8	11.6	14.96	26.0	612	35.9	1174	743	74.6	292	138	99.4	40.8	64.4	14.3	61.1

**Table 3:** Diagnostic ratios of PAHs for three moss species and quartz filters over a six-month sampling period.

	Month	ΣLMW/ΣHMW	FL/(FL + PYR)	ANT/(ANT + PHE)	FLT/(FLT + PYR)	BaA/(BaA + CHR)	IP/(IP + BghiP)	BaP/BghiP
	Sep	0.05	0.55	0.11	0.55	0.28	0.54	0.80
	Oct	0.04	0.53	0.12	0.53	0.39	0.53	0.91
**Filter**	Nov	0.01	0.50	0.15	0.50	0.40	0.54	0.82
	Dec	0.02	0.52	0.36	0.52	0.49	0.55	1.11
	Jan	0.03	0.53	0.15	0.53	0.46	0.56	1.02
	Feb	0.02	0.52	0.23	0.52	0.45	0.57	0.95
**Average**		0.03	0.52	0.19	0.52	0.41	0.55	0.94
	Sep	0.35	0.00	1.00	0.00	0.00	0.62	0.01
	Oct	0.54	0.63	0.07	0.63	0.21	0.49	0.58
***S. fallax***	Nov	0.41	0.62	0.07	0.62	0.25	0.49	0.65
	Dec	0.27	0.62	0.07	0.62	0.29	0.53	0.70
	Jan	0.29	0.63	0.06	0.63	0.25	0.46	0.54
	Feb	0.37	0.65	0.06	0.65	0.24	0.49	0.63
**Average**		0.37	0.53	0.22	0.53	0.21	0.51	0.52
	Sep	13.48	0.00	1.00	0.00	0.00	0.67	0.74
	Oct	1.79	0.57	0.05	0.57	0.26	0.49	0.59
***D. polysetum***	Nov	1.28	0.57	0.03	0.57	0.30	0.45	0.50
	Dec	0.65	0.60	0.05	0.60	0.32	0.49	0.69
	Jan	0.63	0.61	0.05	0.61	0.32	0.44	0.57
	Feb	0.51	0.62	0.04	0.62	0.27	0.45	0.45
**Average**		3.06	0.50	0.21	0.50	0.24	0.50	0.59
	Sep	0.00	0.00	0.00	0.00	0.00	0.00	0.00
	Oct	0.48	0.63	0.04	0.63	0.21	0.53	0.64
***P. schreberi***	Nov	0.33	0.62	0.04	0.62	0.22	0.55	0.66
	Dec	0.25	0.60	0.06	0.60	0.29	0.53	0.75
	Jan	0.25	0.60	0.06	0.60	0.28	0.54	0.77
	Feb	0.27	0.61	0.06	0.61	0.20	0.51	0.67
**Average**		0.26	0.51	0.04	0.51	0.20	0.44	0.58

## Data Availability

The data presented in this study are available in this study only and in the supporting information in Supplementary Materials.

## Supplementary Materials

The following supporting information can be downloaded at: https://www.mdpi.com/article/10.3390/molecules30194009/s1, Figure S1. Trends in cumulative contamination changes for six HMW compounds, including: (a) BaP, (b) DBA, (c) BbF, (d) BkF, (e) IP, and (f) CHR in six months; Figure S2. Combined diagram illustrating the relationship between solar radiation (MJ/m^2^/day) and cumulative PAH concentrations (μg/g) in three moss species over six months. Bar heights represent monthly solar radiation, while lines depict cumulative PAH uptake in *S. fallax*, *D. polysetum*, and *P. schreberi*; Figure S3. Combined diagram showing the relationship between: (a) minimum temperature (°C), and (b) maximum temperature (°C) and cumulative PAH concentrations (μg/g) in three moss species over six months. Yellow bars represent monthly (a) minimum, (b) maximum temperature, while the lines illustrate cumulative PAH uptake in *S. fallax*, *D. polysetum*, and *P. schreberi*; Table S1. Regression relationships between three mosses and filters with weather data on 6 months; Table S2. Meteorological parameters for the duration of the experiment; Table S3. A detailed description of the procedure for preparing moss and filter samples for measuring PAHs.
