# Supplementary material for: Comparative (Bio)monitoring of Airborne PAHs Using Mosses and Filters
Source: Molecules. 2025 Oct 7;30(19):4009. doi: 10.3390/molecules30194009 (PMC12526000; doi:10.3390/molecules30194009)
Supplement: Supplementary file 1 [file molecules-30-04009-s001.zip › molecules-3874529-supplementary.pdf]

Małgorzata Rajfur[1,*], Paweł Świsłowski[1,*], Tymoteusz Turlej[2], Oznur Isinkaralar[3], Kaan Isinkaralar[4], Sara Almasi[5], Arianna Callegari[5], Anca-Iulia Stoica[6]

Comparative (Bio)monitoring of Airborne PAHs Using Mosses and Filters – Supplementary Materials

[1] *Institute of Biology, University of Opole, Opole, Poland*

[2] *Department of Power Systems and Environmental Protection Facilities, AGH University of Science and Technology Cracow Sciences, Cracow, Poland*

[3] *Department of Landscape Architecture, Faculty of Engineering and Architecture, Kastamonu University, Kastamonu, Turkiye*

[4] *Department of Environmental Engineering, Faculty of Engineering and Architecture, Kastamonu University, Kastamonu, Turkiye*

[5] *Department of Civil Engineering and Architecture, Faculty of Engineering, University of Pavia, Pavia, Italy*

[6] *Institute of Sanitary Engineering and Water Pollution Control (SIG), University of Natural Resources and Life Sciences, Vienna, Austria*

[*] Corresponding authors: rajfur@uni.opole.pl, pawel.swislowski@uni.opole.pl

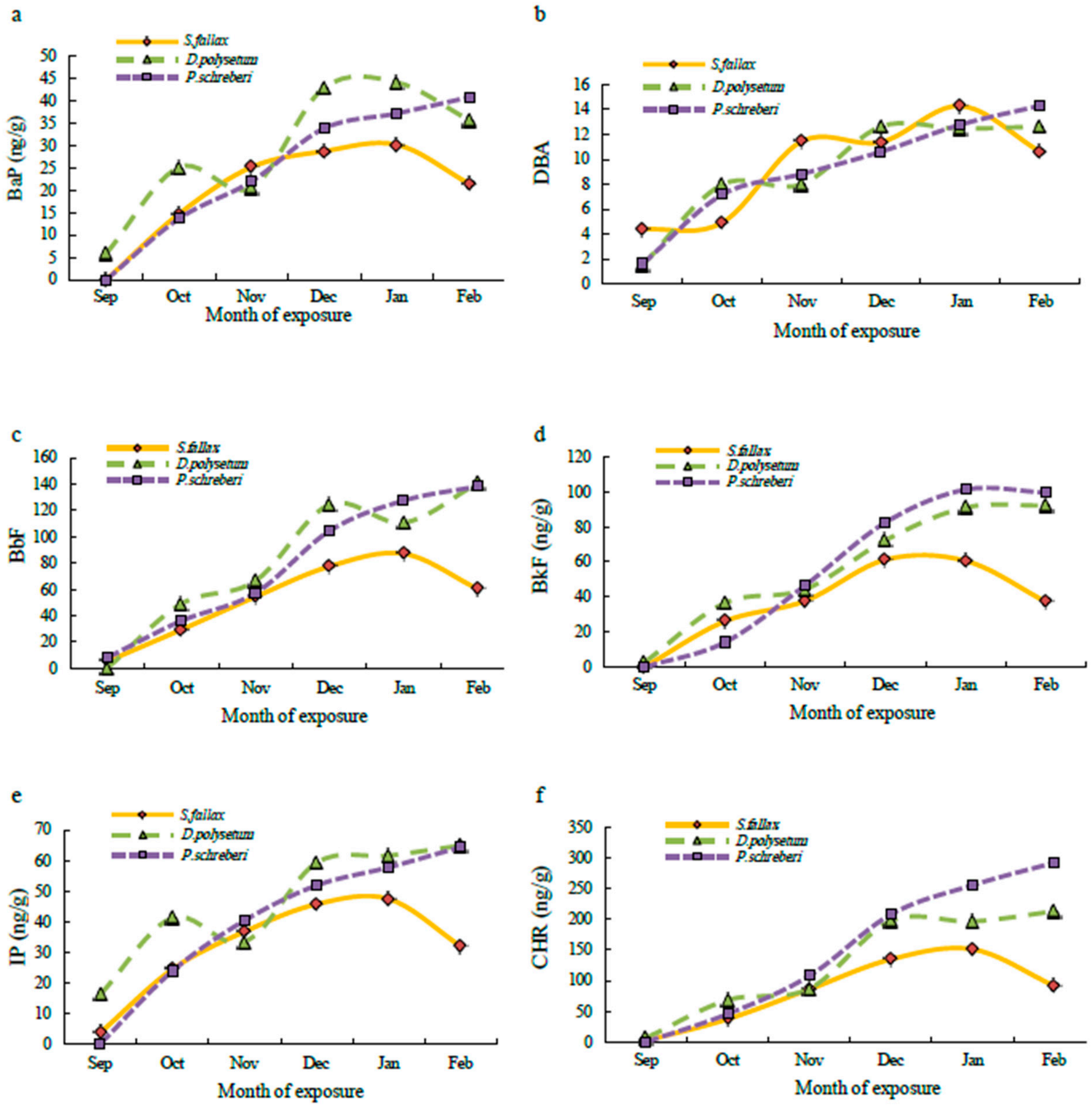

Figure S1. Trends in cumulative contamination changes for six HMW compounds including : a) BaP, b) DBA, c) BbF, d) BkF, e) IP, and f) CHR in six months

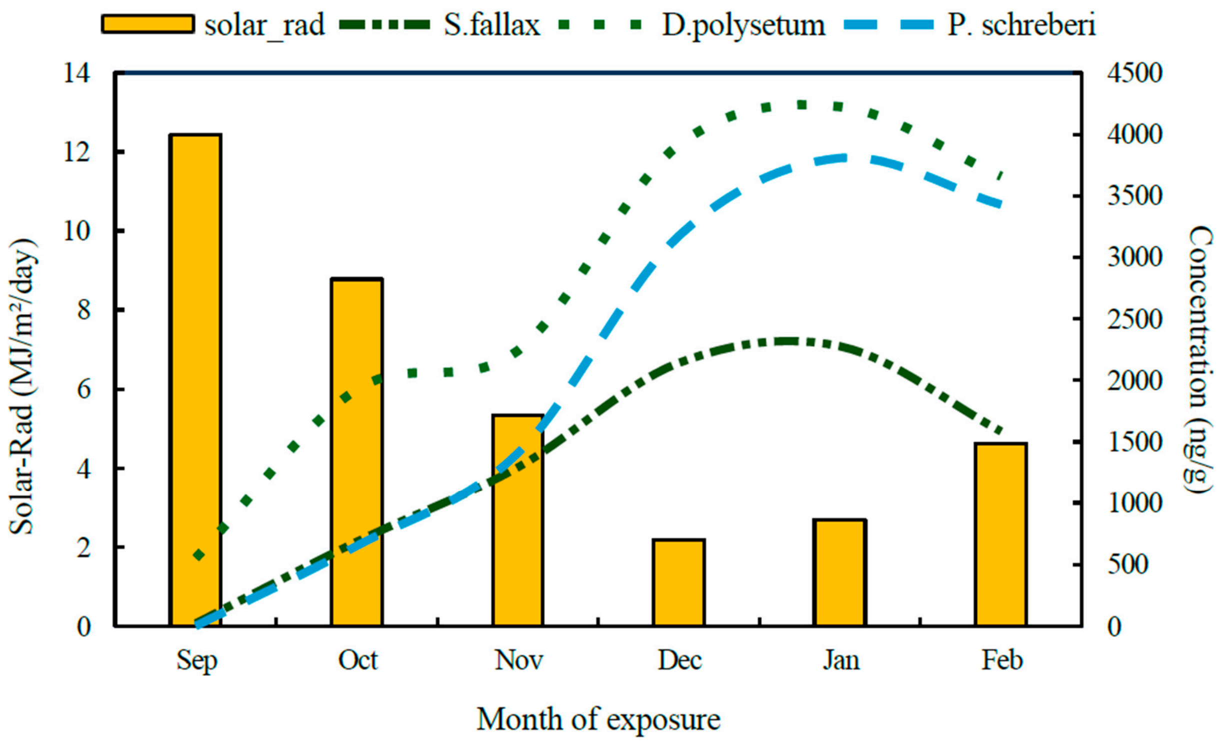

Figure S2. Combined diagram illustrating the relationship between solar radiation (MJ/m²/day) and cumulative PAH concentrations (μg/g) in three moss species over a six-month period. Bar heights represent monthly solar radiation, while lines depict cumulative PAH uptake in *S. fallax*, *D. polysetum*, and *P. schreberi*.

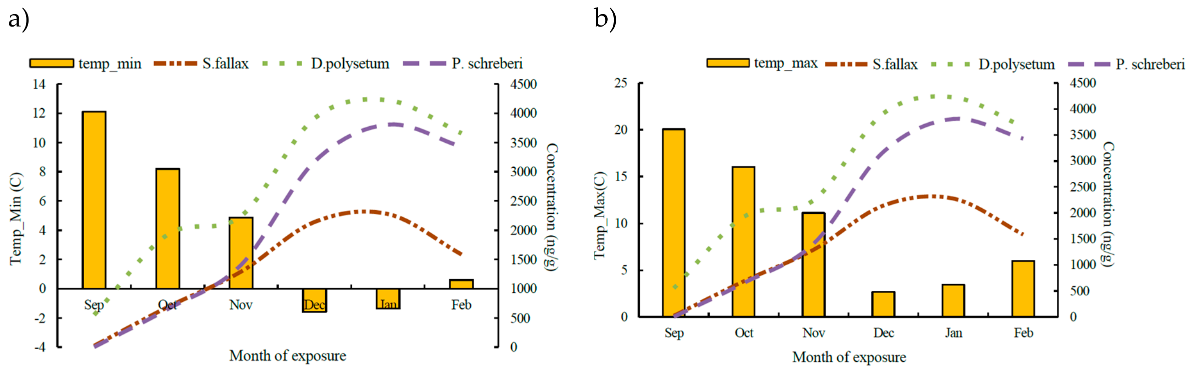

Figure S3. Combined diagram showing the relationship between: a) minimum temperature (°C), and b) maximum temperature (°C) and cumulative PAH concentrations (µg/g) in three moss species over a six-month period. Yellow bars represent monthly a) minimum, b) maximum temperature, while the lines illustrate cumulative PAH uptake in *S. fallax*, *D. polysetum*, and *P. schreberi*.

Table S1. Regression relationships between three mosses and filters with weather data on 6 months.

Treatment	Precip	Solar_Rad	Temp_Max	Temp_Min	Temp_Mean	Wind_Speed
**Filter**	-	y=359545x-26658	-	-	-	-
S. fallax	-	y=2641x-217	y=2522x-120	y=1917x-153	y=2267x-136	-
D. polysetum	-	y=4834x-346	y=4697x-196	y=121x-252	y=4282x-223	-
P. schreberi	-	y=4314x-372	y=4240x-218	y=212x-279	y=3373x-247	-

The linear regression equations used the form y = ax + b with x representing meteorological variables and y representing total PAH concentrations (μg/g). The results show no correlations between variables since dashes (–) are used in the table.

Table S2. Meteorological parameters for the duration of the experiment

Month	Precipitation	Solar rad	Max Temp	Min Temp	Mean Temp	Wind speed
**Sep-2021**	3.06	12.44	20.06	16.11	12.11	2.36
**Oct-2021**	1.16	8.78	16.03	12.04	8.20	3.13
**Nov-2021**	0.76	5.34	11.11	7.74	4.87	3.33
**Dec-2021**	1.73	2.19	2.66	0.82	-1.58	2.76
**Jan-2022**	1.76	2.69	3.47	0.97	-1.35	3.65
**Feb-2022**	2.41	4.61	5.96	3.35	0.60	4.52

Meteorological parameters recorded during the six-month monitoring period. Monthly values include precipitation (mm), solar radiation ($MJ/m^2/day$), maximum, minimum, and mean temperature (°C), and wind speed (m/s), used to evaluate climatic influences on PAH accumulation.

Table S3. A detailed description of the procedure for preparing moss and filter samples for measuring PAHs

**1. Samples preparation for PAHs determination**	**2. Analytical Procedure for PAHs measurement**
A standardized two-step solvent extraction method was implemented for the samples where 0.5 grams of moss went into a clean Erlenmeyer flask while a complete quartz filter obtained its own clean Erlenmeyer flask. Each filter sample was weighed and treated separately. The experiment required 50 mL of acetone solution and 5 μL of PAH-Mix 24 standard added to each of the flasks. A horizontal shaking technique	The evaluation and measurement of PAH compounds used a gas chromatograph (Agilent 6890N) together with an inert mass selective detector (Agilent 5975 MSD) and integrated an automatic liquid injector (Gerstel MPS2). The separation of compounds occurred on an Agilent HP-5MS capillary column which measured 30 m in length and had an inner diameter of 0.25 mm and 0.25 μm film thickness. This column was

lasted thirty minutes before adding 50 mL of cyclohexane for additional shaking to proceed for an additional sixty minutes. The extracts were decanted through separate funnels; for moss samples, the funnel was plugged with quartz wool to prevent small moss particles from passing through. The solid residue in both samples was washed with an additional 50 mL of cyclohexane, and the washings were decanted again. The combined extracts were washed twice with 400 mL of distilled water to remove acetone. The organic phase of each sample was then dried over anhydrous sodium sulfate packed in a glass column, which was rinsed with 15 mL of cyclohexane. Finally, the extracts were concentrated to 1 mL using a rotary evaporator, and in case of necessity, gentle nitrogen evaporation was applied. The rotary evaporator was set at a temperature of 40°C with a rotation speed of 235 rpm to ensure the efficient concentration of the extracts.

coated with 5% phenyl methyl polysiloxane. The system used helium carrier gas at a fixed flow rate of 1.5 mL/min resulting in an average linear velocity of 45 cm/s.

The oven temperature began at 80°C for 10 minutes before increasing at 25°C/min to 200°C and continued to 300°C at 5°C/min for 2.2 minutes of holding time. The maximum temperature allowed in the oven reached 325°C. The total runtime was 37.00 minutes. The system accepted 10 μL syringes for splitless injection mode. The liquid solution entered the system using a 1.0 μL volume at a speed of 100 μL/s. The instrument filled up to 5.0 μL using three strokes at a speed of 5.0 μL per second. The procedure did not involve air volume below the sample and splitless mode was not enabled.

The inlet temperature was maintained at 300°C, and the transfer line to the MSD was held at 300°C. The system operated in selected ion monitoring (SIM) mode with electron ionization (EI) at 70 eV. Calibration was performed using PAH-Mix 9 as an external standard, and PAH-Mix 24 (deuterated PAHs) was added to each sample prior to extraction to serve as an internal standard and correct for matrix effects. The analytical parameters and GC-MS settings followed previously validated protocols for PAH analysis in airborne and moss samples.

108
